# Analysis of Spatial and Biochemical Characteristics of In Vitro Cariogenic Biofilms

**DOI:** 10.7759/cureus.53871

**Published:** 2024-02-08

**Authors:** Poojitha Kumaran, Ramya Ramadoss, Sandhya Sundar, Suganya Panneer Selvam, Bargavi P, Pratibha Ramani

**Affiliations:** 1 Dentistry, Saveetha Dental College and Hospitals, Chennai, IND; 2 Oral Pathology and Oral Biology, Saveetha Dental College and Hospitals, Chennai, IND; 3 Nanotechnology, Saveetha Dental College and Hospitals, Chennai, IND; 4 Oral and Maxillofacial Pathology, Saveetha Dental College and Hospitals, Chennai, IND

**Keywords:** fourier transform infrared spectroscopy (ftir), field emission scanning electron microscope (fesem), microbial ecosystem, biofilm, dental caries

## Abstract

Background

Dental caries is the most common bacterial disease of calcified tissues of teeth. Cariogenic biofilms formed on the tooth surface secrete organic acids and thus result in demineralization. Delving into the depth of biofilms is crucial to understand the pathogenic mechanisms and design improved therapeutic approaches. The aim of the study is to analyze the spatial and biochemical characteristics of cariogenic biofilms.

Materials and methods

Pulp tissue samples sourced from freshly extracted third molars were incubated with oral cariogenic bacteria namely *Streptococcus mutans*,* Staphylococcus aureus*,* Escherichia coli*,* Entamoeba faecalis*, and* Candida albicans *to form the biofilm. Spatial assessment of biofilms was done under FESEM (field emission scanning electron microscope, JSM-IT800, JEOL, Tokyo, Japan). FTIR (Fourier transform infrared spectroscopy, Alpha II, Bruker, Germany) spectra were assessed for chemical molecular interactions in 24- and 48-hour time periods.

Results

Morphological assessment with FESEM revealed rapid growth and aggregation within a short time period. FTIR spectra to analyze chemical constituents of biofilm presented with varied peaks of water, amide A, amide I, water, lipids, and phospholipids.

Conclusion

Further validation with more advanced imaging for an extended time period is vital to derive better conclusive evidence.

## Introduction

Dental caries is the most common infectious disease of mankind, and the worldwide prevalence rate of dental caries accounts for 65.6% to 72.1%. Humans are susceptible to this disease in all stages of their life. Dental caries is a complex interplay of a multitude of factors involving the host and the causative agent [1]. Dysbiosis of the microbial environment commonly leads to the formation of dental biofilm. Inhibiting the cariogenic microorganisms, using an anti-biofilm agent, and maintaining a healthy sugar intake are all effective preventive measures [2,3].

The creation of acidic pH niches by the microbial colonies is referred to as cariogenic biofilms. They are formed within minutes of cleaning or brushing the teeth, and, therefore, it is important to take the necessary steps to prevent the formation of caries. The topical use of re-mineralizing agents is routinely used in the prevention of dental caries [4-6].

To effectively analyze the nature and prevalence of caries, it is important to understand the spatial and biochemical characteristics of the cariogenic biofilms. Different microorganisms and their colonies respond with different growth patterns and thus the complexity of the biofilm is increased. These infectious biofilms are also imperturbable against host immune cells and difficult to fight against host immune cells and antibiotic therapies [7]. The matrix binding of the biofilms and the catalase secreted by the bacterial biofilm make it impossible for the antibodies to penetrate and further increase the impenetrability [8,9].

It should also be noted that microorganisms are not randomly arranged but exhibit specific spatial characteristics. This depends on the microenvironment in which they thrive and the congenital capabilities of the microorganism. Distinct spatial patterns of cells have also been observed in biofilm communities that have been experimentally established. In the same experiments, their metabolic activities, antimicrobial tolerance, and processes of evolution have also been evaluated [9,10]. Understanding the spatial and biochemical characteristics in terms of functional groups of cariogenic biofilms that are crucial to establishing improved therapeutic management has not been done before. Hence, this study aims to analyze the spatial and biochemical characteristics of the cariogenic biofilms.

## Materials and methods

Sample preparation

In vitro analysis of cariogenic activity was evaluated on human pulp tissue samples from freshly extracted third molars derived from the extracted tooth repository, Saveetha Dental College and Hospitals, Chennai, Tamil Nadu, India. The pulp tissue was extirpated after intentional access preparation under a controlled temperature of 180°C with a barbed broach. The extirpated pulp was further subjected to in vitro formation of cariogenic biofilm, followed by its characterization.

 In vitro cariogenic biofilm

Microorganisms used for the formation of cariogenic biofilms were *Streptococcus mutans* strain (NCTC 10449), *Enterococcus faecalis* (ATCC 29212), *Escherichia coli *(ATCC 25922), *Staphylococcus aureus* (ATCC 29213), and *Candida albicans *strain (SC5314). Biofilm development was done by using cultures of bacteria maintained at −80°C. They were incubated in a 96-well plate for a period of 24 hours at 37°C in brain heart infusion media, and incubation was maintained for a period of 24 and 48 hours at room temperature and further maintained in an incubator at optimal conditions. To ascertain biofilm formation, each well was filled with tryptic soy broth, and cell suspension was taken to assess biofilm development. Wells were air-dried, washed with distilled water, and stained with safranin dye. Biofilms were then studied for 24 and 48 hours.

The spatial assessment was done under FESEM (field emission scanning electron microscope, JSM-IT800, JEOL, Tokyo, Japan), which employs field effect guns to increase spatial resolution by concentrating low- and high-energy electrons at low electrical potentials (roughly 0.02 to 5 kV). Biofilms were mounted on the cover slip at two magnifications, 10 µm and 50 µm, after incubation. The sections were dehydrated with 70% ethyl alcohol for 10 seconds before being dyed with nitrogen gas. The sections were sputter-coated with platinum for 30 seconds to induce conductivity before dyeing until the critical point to prepare them for FESEM analysis. Furthermore, pictures were taken at an acceleration voltage of 3 kV and projected. The chemical interactions and the functional groups were assessed using FTIR (Fourier transform infrared spectroscopy, Alpha II, Bruker, Germany) [10].

## Results

**Figure 1:**
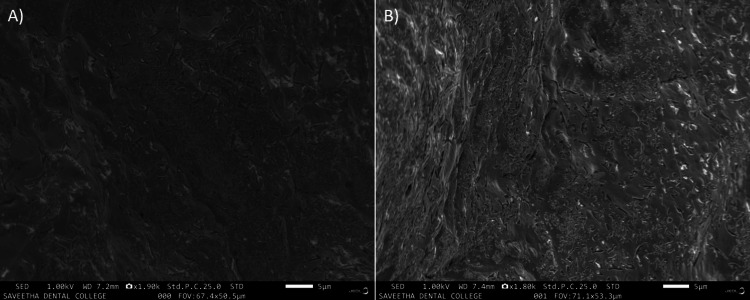
(A, B) SEM image of pulpal tissue after 24 hours of incubation exhibiting minimal colonization of microbe SEM, scanning electron microscopy

The tissue samples were incubated for a duration of 24 hours, and within that limit, a minimal colony of *Streptococcus mutans* strain (NCTC 10449), *Enterococcus faecalis* (ATCC 29212), *Escherichia coli* (ATCC 25922), *Staphylococcus aureus *(ATCC 29213), and *Candida albicans* strain (SC5314) were observed to be formed, as shown in Figures 1A, 1B).

**Figure 2:**
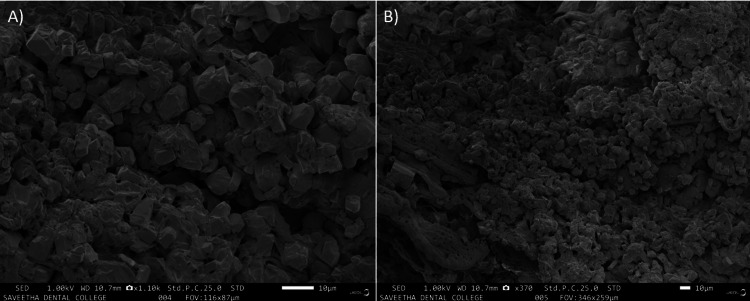
(A, B) SEM image of pulpal tissue at 48 hours of incubation exhibiting intense colonization of microbes SEM, scanning electron microscopy

As shown in Figures 2A, 2B, in the extirpated pulp, after 48 hours, the organisms discovered that the particles had combined to form separate colonies, and it was observed that their size had increased.

**Figure 3:**
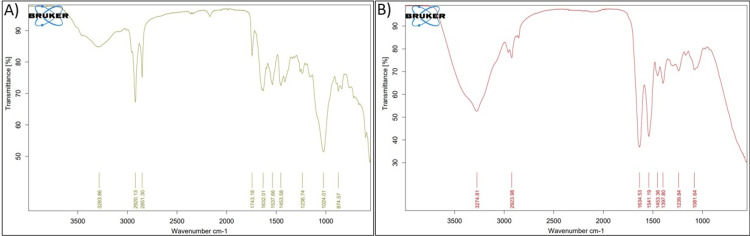
FTIR analysis of the biochemical characteristics of cariogenic biofilm: (A) 24-hour biofilm and (B) 48-hour biofilm FTIR, Fourier transform infrared spectroscopy

Analysis of FTIR spectra of biofilms on pulp tissue revealed chemical molecular interactions during different time periods. Structural changes in the biofilms after 24 and 48 hours of incubation (Figures 3A, 3B) indicated that the intensity of all peaks was less. It showed that water, amide A, amide I, lipids, and phospholipids were all present in the range of 2,950-2,960, 1,700-1,600 of (C=O), 20% of (C-N), 80% of H_2_O, and 1,080-1,070 of (C-C), respectively. Skeletal vibration was found to be associated with D-glucose's anomeric structures. Tryptophan-specific D-glucan anomeric ring vibrations were also observed.

## Discussion

Biofilms were first described in the year 1978 as aggregates of microbes that cling to a surface. They gathered a lot of curiosity and interest due to the complexity and challenges that incurred during management. They were ascribed to about two-thirds of the infection-related diseases. Description of biofilm also evolved along with other significantly contributing factors such as the attachment surface, matrix of host, and bacteria. Another critical property of biofilms is that they are mostly unresponsive to the currently available antibiotic treatments. Continuous attempts to target them are in the fray with less success [11,12].

Dental caries, which is currently the world's most widespread microbial disease, also occurs due to the formation of biofilms. Numerous studies are ongoing to prevent the formation of caries; however, to date, no method can completely prevent the disease [2,3]. Dental biofilm accumulates on the tooth surface as an everyday event, and the matrix formed from extracellular polymers assists in the host's defense to prevent bacterial colonization, creating an oral microbial balance [13,14]. The formation and growth of cariogenic (caries-related) biofilms is due to a breakdown in homeostasis in the oral microenvironment. The current study aimed to evaluate the complexities behind cariogenic biofilms since understanding the intricacies of cariogenic biofilm formation is crucial for deriving effective treatment strategies. The bacteria were chosen due to their association with dental plaques and caries.

Ultrastructural characterization of cariogenic biofilms has been attempted with advanced microscopic imaging. Conventional scanning electron microscopy and field emission scanning electron microscopy offer a synergistic leverage of higher magnifications ranging from up to 30,000× and a resolution of 100 nm [15]. This precedence was evidenced in our FESEM study also with evidence of enhanced microbial colonization in the in vitro cariogenic biofilm in different periods (Figures 1, 2). However, the inherent disadvantage of electron microscopy is that sample processing can result in dehydration and loss of topography with the destruction of the matrix, leading to contraction of the biofilm [15].

Most of the literature reports on cariogenic biofilms discuss histological, microscopic, and mechanical aspects only. Biochemical characterization is more fundamental to aid in better understanding biofilm properties [16,17]. FTIR is one such potent analytical method that renders explicit details on the molecular and chemical configuration of organic components in tissue samples. The key advantage is that it is rapid, non-invasive, and stain- and label-free. FTIR assessment on carious dentin spectra demonstrated peaks suggestive of mineralized dentin with absorbance bands indicative of hydroxyapatite [18,19].

The organic composition of infected zones exhibited more amide peaks due to the presence of increased collagen. Raman spectroscopic analysis of Pseudomonas species biofilms has assisted in the detection of nucleic acids, carbohydrates, proteins, and extracellular matrix proteins. Furthermore, it was also confirmed that complex biofilms with more bacteria showcase a larger biomass than those constructed with single species [20]. FTIR analysis revealed the presence of enhanced polymeric carbohydrates. The analysis further showed varied amide I and glucan bands with diverse combinations of bacteria, which provides evidence for the degree of caries formation potential (Figure 3). Our results evinced a lot of interest in terms of significant variation of biochemical characteristics with time duration, unlike other studies. This pronounced alteration within a shorter period has to be considered for further evaluation and validation.

Limitations of the study

The study needs to be further validated using advanced imaging systems to explore the spatial configuration of the biofilm and strengthen the pathogenic mechanisms with gene expression studies.

## Conclusions

This study revealed that there was intense colonization and co-aggregation of the bacterial colony on the pulpal tissues within a short time. Functional groups involved were also demonstrative of tremendous alterations with increase in time. Further validation with more advanced imaging for an extended time period is vital to derive better conclusive evidence.
